# Hydrogen inhalation ameliorates lung inflammation in mice with asthma

**DOI:** 10.1186/s40001-025-03296-7

**Published:** 2025-10-27

**Authors:** Hongchang Li, Xia Qi, Hongyan Xu, Rong Zhou, Xiaochun Liu, Lei Li, Cuiping Hu, Chunli Dai

**Affiliations:** Qingdao Special Servicemen Recuperation Center of PLA Navy, Qingdao, 266071 Shandong Province China

**Keywords:** Hydrogen, Asthma, Ovalbumin, Lung, Inflammation

## Abstract

Asthma, a prevalent noninfectious chronic disease characterized by type II inflammation, features airway hyperreactivity, bronchoconstriction, and airway remodeling, ultimately causing widespread airway narrowing. Hydrogen has been shown to exhibit antioxidant and anti-inflammatory properties that are beneficial for a range of diseases. This study initially investigated the ameliorative effects of hydrogen gas inhalation on lung inflammation in mice with asthma induced by ovalbumin (OVA). The mice were first sensitized with OVA and subsequently exposed to nebulized 1% OVA via the airway to induce an asthma model. Hydrogen was inhaled for 7 consecutive days as a therapeutic intervention to detect changes in various indicators. Compared with the control treatment, hydrogen inhalation significantly mitigated OVA-induced airway hyperreactivity and inflammation. Hydrogen inhalation attenuated the immune response; decreased the levels of IL-4, IL-5 and IL-13; and further increased the mRNA expression levels of Treg-associated cytokines, namely, IL-10 and TGF-β1, thereby bolstering the body's inflammatory resistance mechanisms. In addition, it also reduced total serum IgE levels and malondialdehyde (MDA) production and increased superoxide dismutase (SOD) secretion in lung tissue. The histological analysis of lung tissues revealed that OVA induced prominent inflammatory cell infiltration and cell proliferation in the alveolar wall, which were markedly ameliorated in the hydrogen-treated group, indicating reduced pathological damage. These findings indicate that hydrogen inhalation effectively suppresses OVA-induced asthma, leading to a substantial improvement in associated lung inflammation, and that the inhalation of hydrogen may be a more feasible approach for future asthma treatment strategies.

## Introduction

Asthma, a prevalent chronic airway disease marked by chronic inflammatory responses, affects approximately 300 million individuals globally, resulting in a significant mortality rate of 250,000 annually [1, 2]. Asthma is characterized by recurrent and often variable symptoms, such as wheezing, breathlessness, chest tightness, and cough, which may worsen at night or during physical activity, significantly impacting patients’ quality of life, posing substantial health risks, and thereby constituting a significant global health, economic, and societal burden [3, 4]. The global prevalence of asthma has exhibited an increasing trend, which has been associated with factors such as urbanization, lifestyle changes, and environmental pollution [5]. This increase is particularly evident among individuals with a family history of asthma; those suffering from comorbidities such as allergic rhinitis, allergic conjunctivitis, and eczema; and those with obesity and smoking habits [6]. Currently, asthma management encompasses a multifaceted approach, including pharmacological interventions, lifestyle modifications, psychological support, breathing exercises, and immunotherapy [7]. Asthma management involves pharmacological interventions, yet the effectiveness of these approaches is often constrained by challenges, including adverse effects of long-term medication use, suboptimal patient adherence, and the persistent influence of environmental triggers [8–10]. Hydrogen, an invisible gas, has attracted increasing attention due to its range of biological effects, including anti-inflammatory, antioxidant, and anti-apoptotic properties [11]. In recent years, advancements in hydrogen medicine have led to extensive investigations into its potential role in managing respiratory diseases, particularly asthma [12]. From an anti-inflammatory perspective, hydrogen gas—especially when administered via inhalation—has demonstrated promising therapeutic effects on airway inflammation [13]. It can reduce oxidative stress and inhibit the release of inflammatory cytokines, thereby improving airway patency and alleviating symptoms such as dyspnea and cough [14]. Additionally, hydrogen exhibits immunomodulatory capabilities that may help balance immune responses and regulate immune cell activity [15], potentially reducing hypersensitivity reactions and decreasing the frequency of asthma exacerbations.

However, it is important to note that current research on hydrogen therapy remains largely experimental, with most evidence derived from preclinical studies and animal models. The clinical applicability of these findings is still uncertain, and well-designed human trials are limited. Furthermore, asthma is a highly heterogeneous disease with diverse endotypes and phenotypes, which may influence how patients respond to hydrogen therapy. Future studies should therefore focus on elucidating the mechanisms of hydrogen's action across different asthma subtypes and on validating these effects in rigorous clinical settings. The advantages of hydrogen therapy in the management of asthma include its high safety profile, convenience of administration, cost-effectiveness, and excellent tolerability. Therefore, the objective of the present study was to investigate the ability of hydrogen gas inhalation to ameliorate lung inflammation in mice with asthma induced by ovalbumin (OVA) and explore its therapeutic value.

## Methods and materials

### Experimental animals

Female C57BL/6 mice, aged 6–8 weeks, were obtained from Yisi Experimental Animal Technology Corporation (Changchun City, Jilin Province, China), maintained in a pathogen-free environment, subjected to a controlled 12 h light/dark cycle, and provided sterile water and irradiated food ad libitum. Prior to any experimental interventions, the mice were acclimated. All experimental procedures conducted in this study adhered to the animal ethics guidelines, ethical clearance number (QDU-AEC-2024595).

### Reagents and measurement

Ovalbumin and alum adjuvant were obtained from Thermo Fisher Scientific. A hematoxylin and eosin (H&E) kit was purchased from Sigma‒Aldrich, and malondialdehyde (MDA) and superoxide dismutase (SOD) detection kits were purchased from Beyotime Biotechnology (Shanghai). ELISA kits for the detection of IL-4, IL-5, IL-13 and IgE levels were obtained from Abcam Biotechnology. The medical hydrogen therapy instrument (QMK-98500Y) and compressed air atomizer were produced by Shenzhen Quantum Hydrogen Biotechnology Co., Ltd.

### Animal experimental protocol

Thirty-two Female C57BL/6 mice were randomly allocated into five groups of eight each: (1) the saline control group, in which mice were sensitized and challenged with saline and exposed to the ambient atmosphere; (2) the 15% H₂-only group, which received no sensitization or challenge but was exposed to 15% H₂; (3) the asthma group, in which mice were sensitized and challenged with ovalbumin (OVA) under the ambient atmosphere; and (4) the OVA + 15% H₂ group, in which mice were sensitized and challenged with OVA and exposed to 15% H₂.

Mice in OVA groups were sensitized via intraperitoneal injection of 100 μL emulsion containing 20 μg OVA and 50 μL alum adjuvant on Days 0 and 14. On Days 24–26, all OVA-challenged groups underwent airway provocation via 30-min inhalation of aerosolized 1% OVA generated by an ultrasonic nebulizer. Hydrogen intervention (15% H₂ in air) was administered via whole-body exposure in a sealed chamber at a continuous flow rate of 4 L/min. Treatment was initiated on Day 21 and delivered daily for 60 min until Day 27. Control mice received saline following the same schedule. All animals were euthanized humanely on Day 28 for sample collection. Following euthanasia via anesthetic overdose, blood samples were collected via the abdominal aorta and immediately transferred into EDTA-coated tubes to prevent coagulation. Plasma was subsequently separated by centrifugation at 3000 rpm for 15 min at 4 °C and stored at −80 °C for further analysis.

### Assessment of airway responsiveness

Airway resistance experiments were conducted using the methodology outlined by Huang et al. [12]. Specifically, airway reactivity in unrestrained, spontaneously breathing mice was evaluated using whole-body plethysmography after challenge with increasing concentrations of nebulized methacholine. The mice were placed in the plethysmograph chamber and initially exposed to nebulized saline for 30 s as a control. The mice subsequently underwent challenges at 20 min intervals with increasing doses of nebulized methacholine (5, 10, 15, 20, 25 and 30 mg/mL) for 30 s each. During each 5-min challenge sequence, the enhanced pause was measured by barometric plethysmography and served as an index of airway obstruction. The results are presented as percentages of the baseline Penh values relative to each methacholine concentration.

### Measurements of BALF cytokine levels and total serum IgG levels

The left main bronchi and right lung lobe were ligated, followed by the insertion of a syringe into the upper tracheal segment to obtain unilateral bronchoalveolar lavage fluid (BALF). Subsequently, 0.3 mL of PBS was administered, and the mouse's chest was gently massaged for approximately one minute to facilitate rinsing [17]. This procedure was repeated three times, and the lavage fluid was subsequently collected. The collected lavage fluid was then centrifuged at 1500 rpm for 10 min at 4 °C and resuspended in 1 mL of PBS.

BALF supernatants from the mice were collected for ELISAs of key indicators of asthma, including IL-4, IL-5 and IL-13, according to the manufacturers’ recommendations. Immediately after BAL, blood was collected to obtain serum for the detection of total IgE, and the left lung was excised and divided into three portions. One portion was preserved in 4% (w/v) formalin for histopathological sectioning; one part was used for RNA extraction to detect the expression levels of relevant mRNAs; and the other was maintained in PBS for the assessment of additional indicators within the lung tissue.

### RT-qPCR detection of Treg-related mRNA expression

The expression levels of Treg-related mRNAs, including IL-10 and TGF-β1, were detected. The preserved lung tissue was processed by adding TRIzol reagent (1 mL per 100 mg of tissue) and thoroughly homogenized using a sterile homogenizer, followed by an incubation on ice for 5 min. Subsequently, 200 µL of chloroform was added to the mixture, which was thoroughly mixed and allowed to stand on ice to ensure the complete dissociation of the nucleoprotein complexes. After centrifugation, an equal volume of precooled isopropanol was added to produce a white RNA precipitate. The precipitate was then gently washed with 75% ethanol and allowed to air dry at room temperature before being dissolved in RNase-free water. Finally, the RNA was reverse transcribed into cDNA according to the instructions provided with the reverse transcription kit. Using the SYBR Green PCR Kit supplied by Thermo Fisher Scientific, real-time qPCR was performed on an ABI-7500 system. Each sample was assayed in triplicate, and the relative gene expression levels were calculated via the 2^−ΔΔCT^ method [18]. The specific primers for GAPDH, IL-10, and TGF-β1 are listed in Table 1 [13, 19].

**Table 1:** The primer sequences of mice IL-10 and TGF-1β

Gene	Sequences	Length (bp)	References
IL-10-F	AGCCGGGAAGACAATAACTG	21	[13]
IL-10-R	CATTTCCGATAAGGCTTGG	19	
GAPDH-F	AGGTCGGTGTGAACGGATTTC	21	
GAPDH-R	TGTAGACCATGTAGTTGAGGTCA	23	
TGF-β1-F	CTCCCGTGGCTTCTAGTGC	19	[18]
TGF-β1-R	GCCTTAGTTTGGACAGGATCTG	22	

### Measurement of MDA content and SOD activity in lung tissues

Following the measurement of the lung tissue weight, an adequate quantity of tissue lysis buffer was added to facilitate thorough homogenization by grinding. The homogenized mixture was then incubated at 4 °C for 30 min to guarantee comprehensive lysis. Centrifugation was subsequently performed to separate and collect the supernatant, which was then used for the assessment of malondialdehyde (MDA) levels and superoxide dismutase (SOD) enzymatic activity.

### Histopathological examination of lung tissues

For histological analysis, the lungs were inflation-fixed intratracheally with 4% paraformaldehyde at a constant pressure to maintain the alveolar architecture, followed by immersion in the same fixative for 24 h before paraffin embedding. Tissue Processing: The tissue was dehydrated in a series of graded ethanol solutions, after which the water was removed, and the tissue was prepared for infiltration with paraffin. Embedding: The dehydrated and cleared tissue was placed in molten paraffin at a temperature of approximately 60 °C. Sectioning: Using a microtome, thin sections (4–6 µm) were cut from the paraffin block. Mounting: Prior to staining, the sections were dewaxed by immersion in xylene followed by a graded ethanol series. Hematoxylin and eosin (H&E) staining was subsequently performed (in accordance with the manufacturer’s protocols) to examine the comprehensive anatomical structure and pathological modifications in the lung tissue.

### Statistical analysis

The collected data were comprehensively analyzed using Prism software (GraphPad), and the results are presented as the means ± standard deviations. One-way ANOVA was performed to assess the significance of differences among the different groups. The levels of significance are denoted as follows: *P < 0.05, **P < 0.01, and ***P < 0.001.

## Results

### Inhalation of hydrogen decreases airway resistance in asthmatic mice

**Fig. 1:**
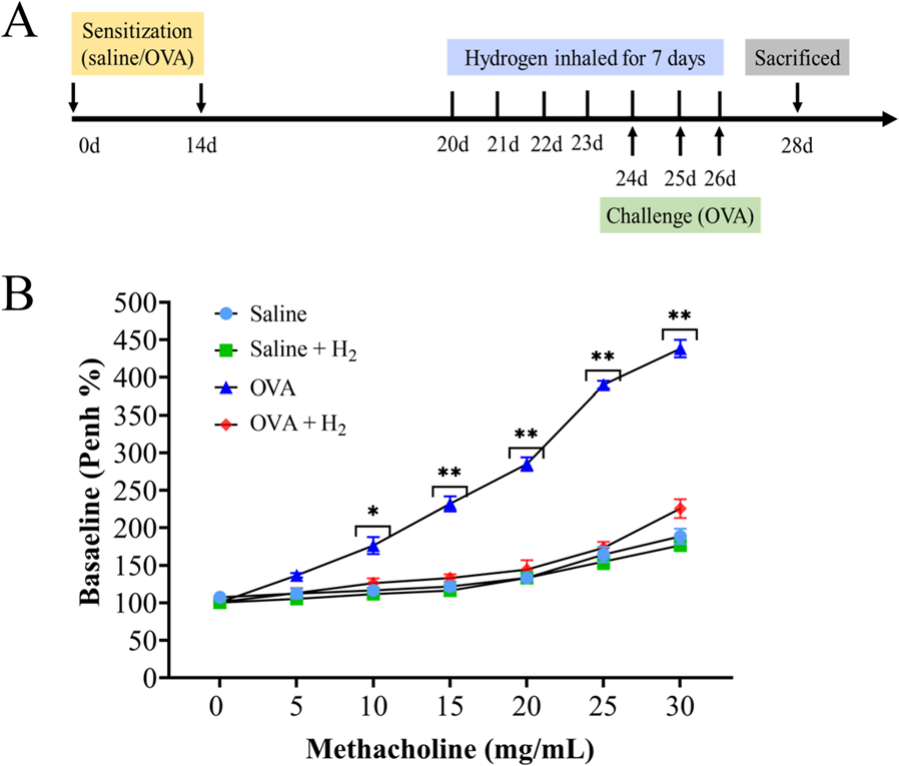
The animal experimental design and effects of hydrogen on airway responsiveness. **A** Animal experimental procedures. **B** Airway responsiveness was assessed using Penh

In this study, an OVA-induced asthma model was employed, and the experimental protocol is illustrated in (Fig. 1A). Airway responsiveness was assessed by measuring the Penh value 24 h after the final challenge. OVA sensitization and challenge resulted in a notable increase in airway responsiveness to inhaled methacholine at doses of 10, 15, 20, 25 and 30 mg/mL for 30 s. Importantly, this increase was substantially mitigated following hydrogen gas inhalation (Fig. 1B).

### Hydrogen inhalation attenuates OVA-induced Th2 responses

**Fig. 2:**
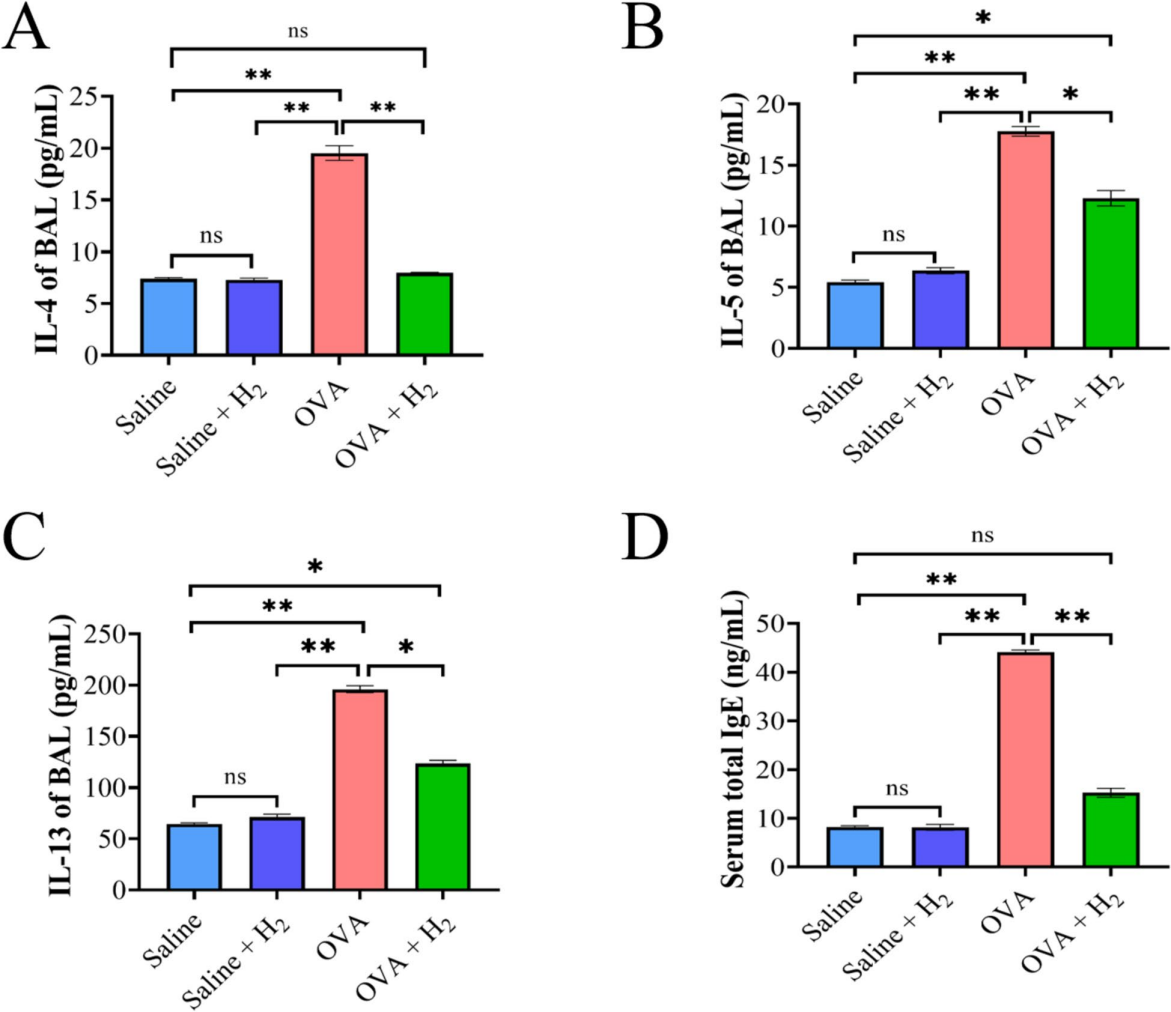
The effect of inhaling hydrogen on OVA-induced Th2 type cytokines and serum IgE. **A** Levels of IL-4 in supernatants of BAL fluids. **B** Levels of IL-5 in supernatants of BAL fluids. **C** Levels of IL-13 in supernatants of BAL fluids. **D** Total serum IgE was detected by ELISA. *P < 0.05 and **P < 0.01

Given that asthma is a widespread noninfectious chronic condition marked by type II inflammation, we assessed the levels of Th2-associated cytokines (IL-4, IL-5, and IL-13) in bronchoalveolar lavage (BAL) fluid and serum IgE concentrations. As anticipated, exposure to OVA markedly increased IL-4 levels, whereas the levels in the hydrogen inhalation group were comparable to those in the control group (Fig. 2A). Compared with saline, OVA significantly increased the IL-5 content (Fig. 2B). IL-13 levels displayed a similar pattern to the levels of IL-5, with hydrogen inhalation exerting a modest suppressive effect on its production (Fig. 2C). Additionally, total IgE levels were analyzed and mirrored the findings observed for IL-4 levels, where OVA exposure significantly increased their levels, whereas hydrogen inhalation significantly decreased them (Fig. 2D).

### Detection of Treg-related indicators (IL-10 and TGF-β1) by qPCR

**Fig. 3:**
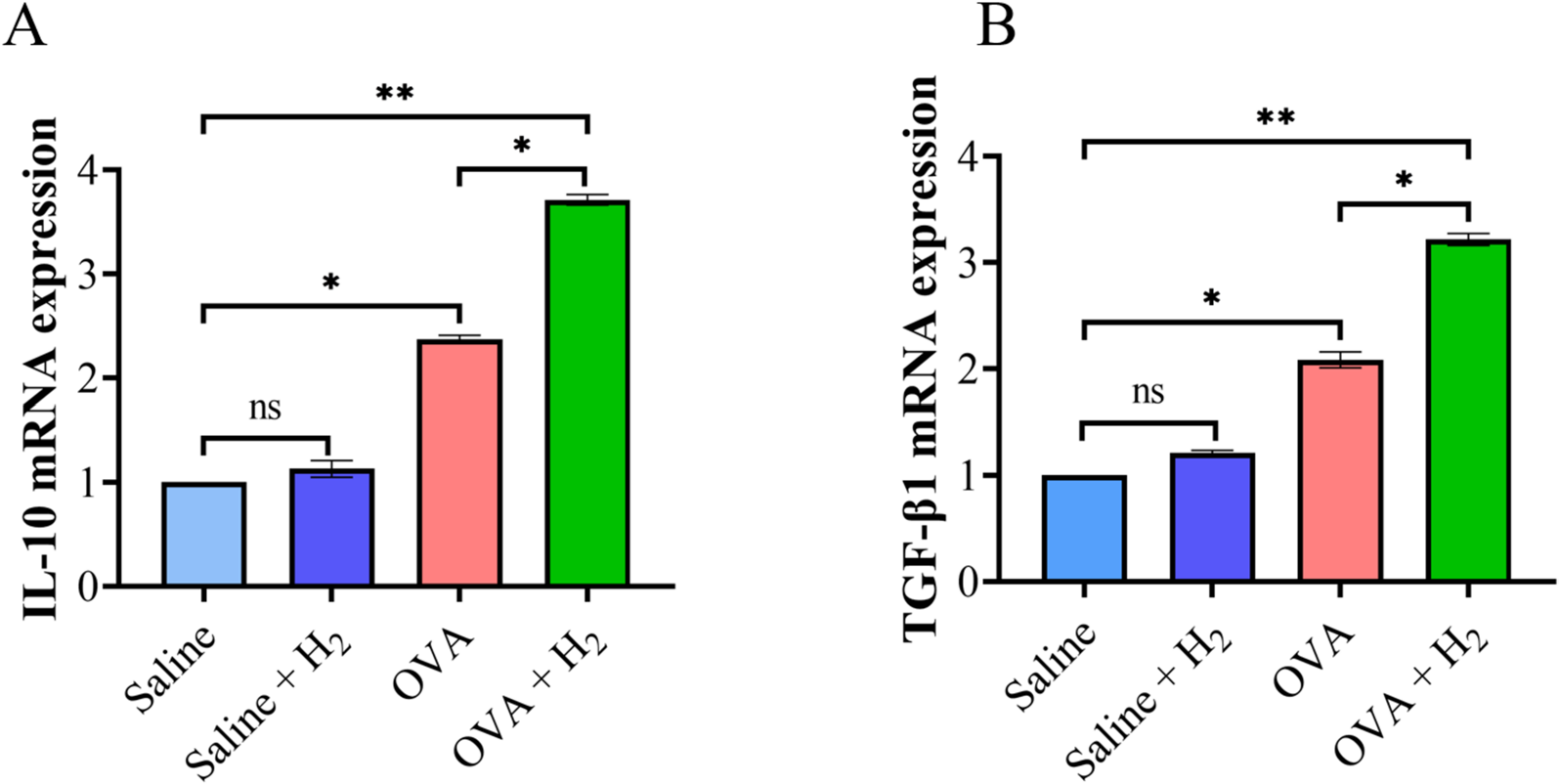
The mRNA expression of IL-10 and TGF-β1 in mice lung tissue. **A** Measurement of IL-10 expression level. **B** Measurement of TGF-β1 expression level. *P < 0.05 and **P < 0.01

qPCR was further employed to investigate the mRNA expression levels of Treg-associated cytokines in lung tissues, and the results revealed the same trend. Compared with the levels in the control group, OVA-induced asthmatic mice presented elevated mRNA expression levels of the anti-inflammatory cytokines IL-10 and TGF-β1 in the lungs. Hydrogen inhalation markedly increased the expression levels of these anti-inflammatory cytokines, suggesting that hydrogen inhalation effectively increases the expression of both IL-10 and TGF-β1, thereby enhancing the body’s anti-inflammatory mechanisms (Fig. 3).

### Hydrogen inhalation decreased MDA levels and increased SOD activity in lung tissues

**Fig. 4:**
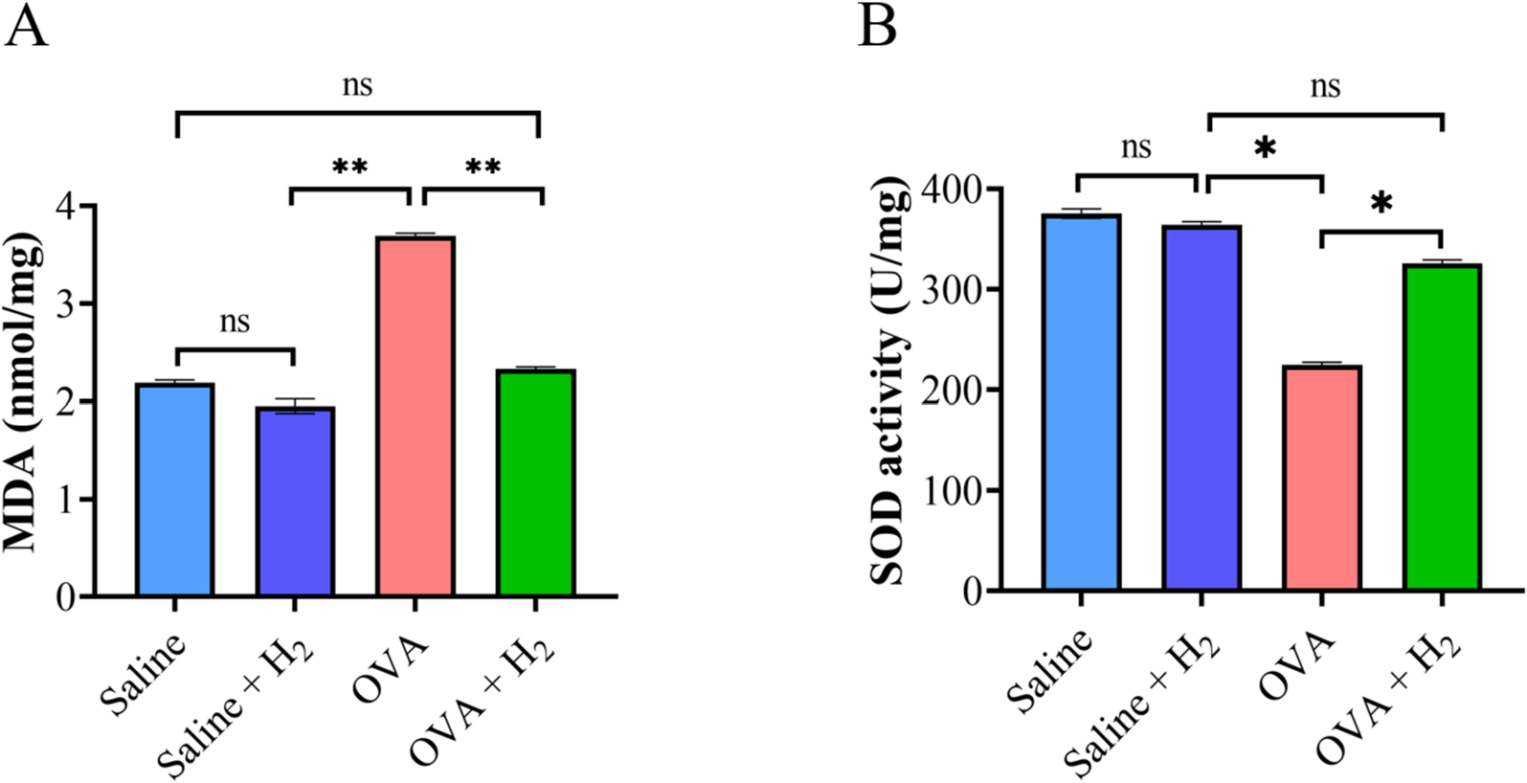
Impact of hydrogen inhalation on MDA levels and SOD activity in lung tissue of mice induced by OVA. **A** Assessment of MDA concentration in lung tissues. **B** Assessment of SOD activity in lung tissues. *P < 0.05 and **P < 0.01

We further substantiated the link between the protective effects of hydrogen gas inhalation and its antioxidant properties by assaying the levels of MDA and SOD. The results revealed that in OVA-induced asthmatic mice, lung MDA levels were markedly elevated, yet they were suppressed to nearly control levels (either the saline or saline + H_2_ group) following hydrogen gas inhalation (Fig. 4A). Concurrently, we observed a decrease in SOD activity in OVA-sensitized and challenged mice, and compared with the OVA group, hydrogen inhalation therapy significantly increased SOD activity, with no difference in the levels compared with those of the control group (Fig. 4B). These findings indicate that hydrogen gas inhalation can safeguard OVA-induced asthmatic mice from oxidative damage.

### Hydrogen gas inhalation ameliorated lung inflammation induced by OVA

**Fig. 5:**
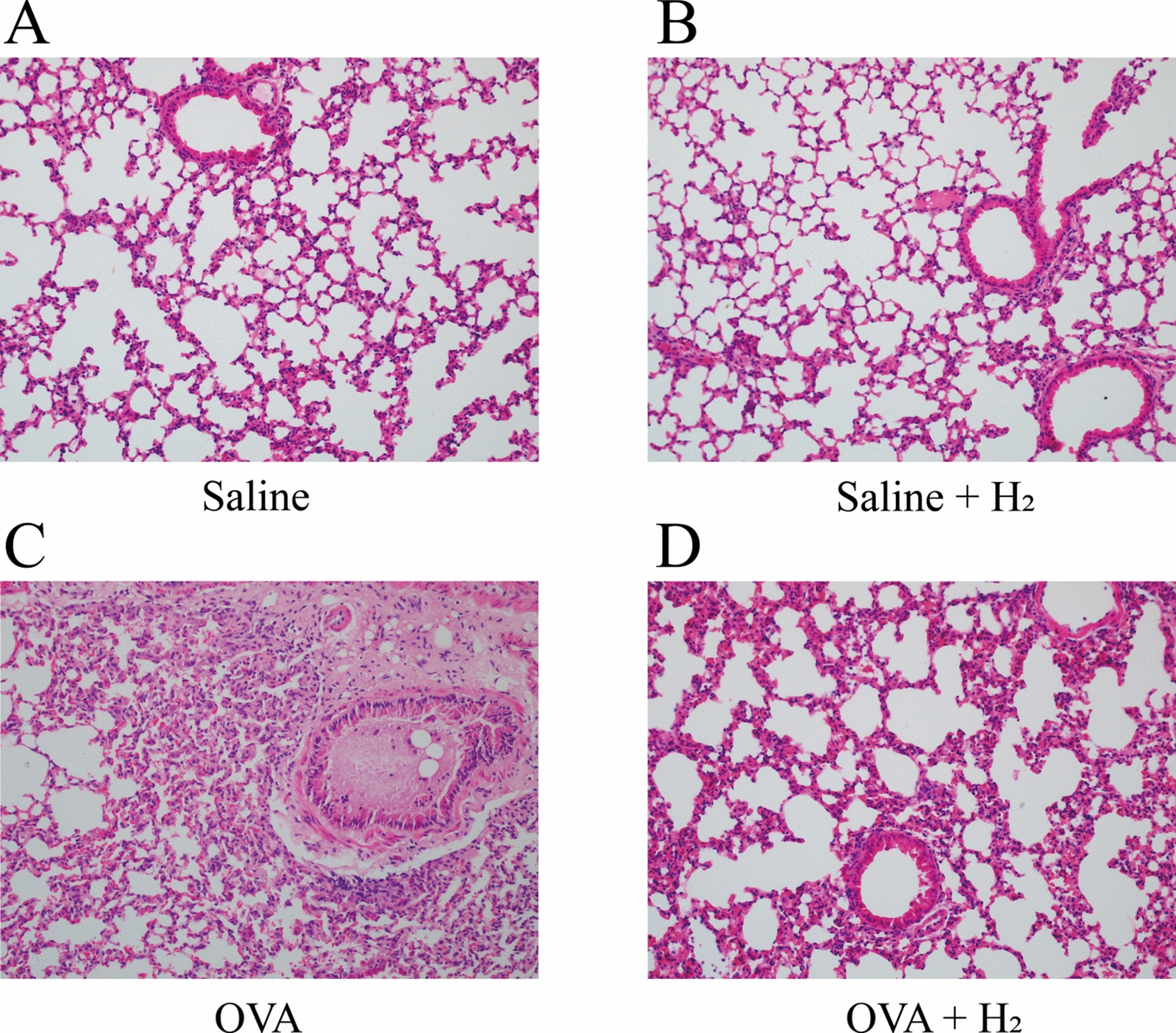
**A** The lung tissue of saline control group.** B** The lung tissue of 15% H₂-only group.** C** The lung tissue of asthma group.** D** The lung tissue of OVA + 15% H₂ group

We embedded lung tissues from mice across experimental groups in paraffin and performed hematoxylin and eosin staining to visually evaluate the potential of hydrogen gas inhalation in alleviating lung inflammation induced by OVA in a murine asthma model. In comparison to the control group, lung sections from OVA-challenged mice showed pronounced pathological changes, including marked thickening of bronchiolar and small bronchial walls, as well as thickening of alveolar septa. These structural alterations were accompanied by substantial inflammatory cell infiltration. In contrast, mice subjected to hydrogen gas inhalation exhibited a significant attenuation of these pathological features. Specifically, hydrogen treatment reduced airway wall thickening and mitigated inflammatory cell infiltration, demonstrating its protective effect against OVA-induced pulmonary inflammation (Fig. 5). These findings indicate a pronounced ameliorative effect of hydrogen gas inhalation on OVA-induced lung injury in asthmatic mice.

## Discussion

Asthma is a complex, chronic noncommunicable disease. It involves overreactive airways, bronchial narrowing, and structural changes, leading to widespread airflow blockage and significant breathing problems. [2, 20]. This inflammatory disease, which is predominantly induced by type II immune-mediated processes, imposes considerable strain on health care resources worldwide, stemming from its widespread prevalence and related morbidity indices [16, 21]. Current treatment approaches, encompassing bronchodilatory agents and glucocorticoids, are targeted at mitigating symptoms and managing disease progression, although they are frequently accompanied by adverse effects and varying levels of therapeutic effectiveness [22]. Due to its antioxidant and anti-inflammatory properties, hydrogen represents a novel therapeutic option that holds promise for a range of diseases, including asthma [11, 23]. Our research revealed that hydrogen gas inhalation markedly alleviated OVA-induced airway hyperresponsiveness and inflammation, suggesting its potential as an adjunct therapy for asthma management.

The OVA-induced murine asthma model represents an acute simulation of allergen-driven asthma, encompassing key features such as airway inflammation metaplasia [24–26]. This model facilitates the exploration of therapeutic interventions under controlled settings, providing valuable insights into mechanisms of action and potential therapeutic efficacy. In our investigation, the choice of indicators, including airway hyperresponsiveness, cytokine concentrations (IL-4, IL-5, and IL-13), IgE titers, and histopathological examinations, was pivotal for evaluating the efficacy of hydrogen therapy. On the other hand, hydrogen inhalation therapy significantly increased the mRNA expression levels of associated cytokines, namely, IL-10 and TGF-β1, thereby bolstering the body’s inflammatory resistance mechanisms, which is consistent with previous research [27, 28]. It is important to note that this study measured cytokine expression at the mRNA level. While the upregulation of IL-10 and TGF-β1 mRNA is a strong indicator, future studies quantifying serum and lung tissue protein levels of these cytokines and incorporating functional T cell assays will be crucial to conclusively validate the role of Treg cell activation in the observed anti-inflammatory effects of hydrogen.

This research recognizes that the utilization of saline as a vehicle for both hydrogen and OVA administration constitutes a limitation in the present study. Although a saline-control group (subjected to sensitization and challenge with saline) was implemented to control for procedural artifacts associated with the immunization and challenge protocols, this setup does not entirely decouple the potential nonspecific effects of the saline vehicle from the specific biological actions attributable to hydrogen. A more rigorous experimental design would entail the inclusion of an additional vehicle-control group receiving saline-only treatments, devoid of any active compounds, to achieve more precise isolation of hydrogen’s contributory mechanisms. Subsequent investigations will integrate such a control cohort to refine the mechanistic interpretation of hydrogen’s therapeutic effects.

In addition, airway hyperresponsiveness, a defining feature of asthma, directly quantifies disease severity. Cytokine profiling and IgE titers provide valuable insights into immune dysregulation, whereas the histopathological analysis visually depicts tissue injury and inflammatory responses. Asthma-induced lung pathology is characterized by the infiltration of inflammatory cells, alveolar wall thickening, and goblet cell metaplasia, all of which contribute to airway constriction and respiratory impairment [29]. These pathological alterations are frequently accompanied by increased oxidative stress, as indicated by elevated levels of malondialdehyde (MDA), a biomarker of lipid peroxidation [30]. Superoxide dismutase (SOD), on the other hand, is a key antioxidant enzyme that scavenges superoxide radicals, thereby mitigating oxidative damage [31, 32]. Our study aimed to evaluate the oxidative stress status in the lungs of asthmatic mice by measuring MDA and SOD levels and to assess the impact of hydrogen therapy on these parameters. The notable decrease in the MDA concentration and increase in SOD activity observed in the hydrogen-treated group indicate that hydrogen effectively mitigates oxidative stress, thereby contributing to its overall anti-inflammatory and protective effects. These observations are consistent with prior research reporting the antioxidant properties of hydrogen in diverse disease models [11–13].

Although this study demonstrates that hydrogen inhalation concurrently ameliorates oxidative stress and inflammation, a pertinent question arises regarding the primacy of these effects. We propose that these mechanisms are inextricably linked rather than existing in a simple causal sequence. The potent antioxidant capacity of hydrogen, by selectively scavenging cytotoxic reactive oxygen species (ROS), directly quenches key signaling molecules that drive the activation of pro-inflammatory pathways, such as NF-κB. Thus, the observed anti-inflammatory effects are both a secondary consequence of reduced oxidative stress and a reflection of an intrinsic anti-inflammatory property mediated through antioxidant action. Furthermore, our finding of enhanced Treg-associated cytokine expression suggests an immunomodulatory role. We hypothesize that by mitigating the oxidative and inflammatory milieu, which is known to be suppressive for Treg function, hydrogen inhalation fosters a microenvironment conducive to Treg-mediated immunoregulation. The subsequent increase in IL-10 and TGF-β1 secretion would then further reinforce the suppression of Th2 responses and promote immune tolerance. While a direct effect of hydrogen on T cell differentiation cannot be ruled out and warrants further investigation, the interconnection between antioxidant and immunoregulatory mechanisms likely forms a positive feedback loop that comprehensively attenuates allergic airway inflammation.

In conclusion, our study showed that hydrogen inhalation effectively suppresses OVA-induced asthma in mice, leading to substantial improvements in associated lung inflammation and pathological damage. By mitigating airway hyperreactivity, reducing the levels of inflammatory cytokines (IL-4, IL-5 and IL-13) involved in the Th2 immune response, and enhancing Treg immune regulation (IL-10 and TGF-β1), hydrogen therapy has emerged as a promising adjunct strategy in asthma management. Furthermore, the observed attenuation of oxidative stress, as indicated by decreased MDA levels and increased SOD secretion, underscores the multifaceted mechanisms of action of hydrogen. The integration of hydrogen therapy into asthma management strategies could provide an approach for this chronic and debilitating disease.

## Data Availability

The data supporting the findings of this study are available on reasonable request from the corresponding author.
